# The Role of Self-reports and Behavioral Measures of Interpretation Biases in Children with Varying Levels of Anxiety

**DOI:** 10.1007/s10578-018-0804-x

**Published:** 2018-04-21

**Authors:** Anke M. Klein, Emmelie Flokstra, Rianne van Niekerk, Steven Klein, Ronald M. Rapee, Jennifer L. Hudson, Susan M. Bögels, Eni S. Becker, Mike Rinck

**Affiliations:** 1 Behavioural Science Institute, Radboud University Nijmegen, PO Box 9104, 6500 HE Nijmegen, The Netherlands; 2 Clinical Psychology, Radboud University Nijmegen, Nijmegen, The Netherlands; 3 Centre for Emotional Health, Macquarie University, Sydney, Australia; 4 Child Development and Education, University of Amsterdam, Amsterdam, The Netherlands

**Keywords:** Interpretation bias, Content-specificity, Children, Spider fear, Social anxiety

## Abstract

We investigated the role of self-reports and behavioral measures of interpretation biases and their content-specificity in children with varying levels of spider fear and/or social anxiety. In total, 141 selected children from a community sample completed an interpretation bias task with scenarios that were related to either spider threat or social threat. Specific interpretation biases were found; only spider-related interpretation bias and self-reported spider fear predicted unique variance in avoidance behavior on the Behavior Avoidance Task for spiders. Likewise, only social-threat related interpretation bias and self-reported social anxiety predicted anxiety during the Social Speech Task. These findings support the hypothesis that fearful children display cognitive biases that are specific to particular fear-relevant stimuli. Clinically, this insight might be used to improve treatments for anxious children by targeting content-specific interpretation biases related to individual disorders.

## Introduction

Anxiety disorders are the most frequent mental disorders in children [1], and they make other disorders and impairments more likely [2]. In light of the extent and burden of childhood anxiety disorders, surprisingly few studies have been reported, compared to the vast number of adult studies. Moreover, approximately 40% of treated children still meet criteria for a clinical disorder after treatment [3]. To date, we know very little about the underlying ‘active’ ingredients of anxiety treatments, but cognitive theories of anxiety disorders have emphasized the critical importance of several cognitive processes in their onset and maintenance [e.g., 4; for a review, see 5]. The central assumption of these theories is that cognitive processes are driven by schemata. Schemata are cognitive structures of associations between knowledge elements that influence perception, attention, interpretation, and memory. In individuals with an anxiety disorder, schemata that are organized around the themes of threat and danger are chronically overactive, and as a result many situations and stimuli are associated with danger and fear [e.g., 6; for a schema-based theory of child anxiety, see 7]. If some stimuli have a particularly strong association with threat and fear (fear-related associations), these stimuli will attract attention quickly (attention bias), their interpretation will be biased towards danger (interpretation bias), and they will be primed in memory (memory bias). These cognitive biases are believed to be content-specific; only stimuli that are associated with threat and fear are processed preferentially. Anxious individuals should therefore only display biased cognitions for stimuli related to their own anxiety [e.g., 4, 8]. For instance, children who are socially anxious should only interpret stimuli that are related to social situations in a negative way (interpretation bias), but not other ambiguous stimuli, such as stimuli related to spiders. Knowing more about the role of content-specificity in childhood anxiety could have important implications for the treatment of anxiety in children, as this might suggest the importance of focusing on disorder-specific cognitions.

Fearful children interpret ambiguous situations in a generally negative way [see 9–11]. However, it is much less clear if fearful children display negative interpretations for their own fear only or if they respond in a negative way to other fear-related stimuli. Some studies have not found any evidence for content-specific interpretations [12–14] whereas other studies have found some evidence for the content-specificity of interpretation biases [15–18]. Several authors have expressed the need for more research on content-specificity in interpretation biases in childhood anxiety [e.g., 9, 17]. Therefore, the main goal of this study was to further explore the content-specificity of interpretation biases in childhood anxiety.

To date, it is not clear why some studies found evidence for content-specificity and others did not. It is possible that these differences are due to different sample characteristics, such as differences in age, gender, levels of anxiety, or differences in the focus of anxiety. However, all studies included both boys and girls and the age of the children across the studies was very similar. Furthermore, significant effects were found in both community samples [15–17] and clinical samples [18], so it seems unlikely that gender, age and levels of anxiety only were responsible for the differences. Alternatively, the difference might be due to the focus of anxiety. For instance, studies focusing on separation anxiety, social anxiety and/or generalized anxiety reported that their sample had high levels of co-morbidity [14, 15, 18]. Co-morbidity of anxieties is one of the most difficult issues in studying specificity, as this makes it more difficult to study the specificity of the biased interpretations of each separate fear. By selecting two relatively independent fears, for instance a physical threat (e.g., spider fear) and a social threat (e.g., social anxiety) this overlap might be reduced [see also 19]. So far, there is only one study that reported on the content-specificity of fear of spiders, but the study included another animal (butterflies) as a reference [17]. In order to be able to replicate the study of Klein et al. [17], but also to be able to compare a physical threat to a social threat, we decided to include children with varying levels of spider fear and/or social anxiety.

Furthermore, differences in findings could also be due to methodological differences. Anxiety is a complex phenomenon, including subjective experience measured by self-reports, behavior (e.g., avoidance behavior), cognitive processes (e.g., interpretation biases) and specific physiological reactions [e.g., 20]. Existing research, at least in children, has mostly used self-reports as the “gold standard” to define levels of anxiety, and, cognitive processes were mostly only related to the self-reports [or an overview, see 21]. This may be insufficient, however, because there are more aspects of anxiety than only self-reported anxiety. For example, previous studies have shown that self-reported symptoms of anxiety only correlate moderately with behavior measured in the real world [22]. As avoidance behavior is one of the key maintaining factors in anxiety, it seems important to not only rely on self-reports, but to also include behavioral measures when studying fear and anxiety. Furthermore, self-reports may reflect more controlled processes while cognitive processes are generally more automatic, and not always open to introspection and self-report, especially in children [see also 23, 24]. Therefore, research on cognitive biases should include both self-reports and behavioral measures, and then relate the biases to both of these measures. We therefore decided to include behavioral measures related to spider fear and social anxiety. For spider fear, we applied a Behavior Avoidance Task (BAT) related to spiders, as previous studies have shown that spider fearful children find it very difficult to approach spiders [25]. For social anxiety, we included a Social Speech Task (SST), based on studies showing that socially anxious children find it very distressing to be the center of attention and to be evaluated on their social performance [22, 26]. The aim of the current study was to add both the BAT and SST to the self-reports, and to examine the relationship between interpretation bias and both behavioral measures and self-reported fear and anxiety.

The goals of this study were threefold. The first goal was to replicate the study reported by Klein et al. [17], and to see if the results concerning interpretation bias in spider-fearful children were similar. They found that children with higher self-reported spider fear and avoidance behavior on the BAT displayed a content-specific interpretation bias for spider threat-related stimuli only, and not to other fear-related stimuli. The second goal was to test whether children with higher levels of self-reported social anxiety and higher anxiety during the SST would show more negative interpretations of ambiguous social threat-related scenarios only, and not of spider threat-related scenarios. The third goal was to compare the independent ability of self-reported fear and interpretation bias to predict avoidance behavior on the BAT, and anxiety during the SST.

First, we expected to replicate the findings of Klein et al. [17] who found evidence for a specific interpretation bias in children with spider fear. Furthermore, based on cognitive theories [e.g., 4, 8] we expected to find evidence for a specific interpretation bias in children with social anxiety. Finally, we expected self-reported fear (direct measure) and interpretation biases (indirect measures) to have unique predictive value for the behavioral task outcomes, because both types of measures tap into distinct processes [27, 28].

## Methods

### Participants

This study was part of a large community-based project about childhood anxiety, for which an unselected sample of children was recruited from regular elementary schools in the Netherlands. After parental active written consent had been granted, a total of 718 children were screened on their levels of social anxiety and spider fear with the SCARED [29] and the SADS-C [25]. Because we were unable to test all children individually, 141 children (101 girls) between 8 and 13 years of age (*M* = 10.0, *SD* = 1.1) were selected, such that (1) levels of social anxiety and spider fear were approximately normally distributed, and (2) approximately the same number of girls/boys scored in the lower and higher regions on self-reported anxiety. The current sample partly overlapped with two other studies that focused on other biases [28, 30]. The Ethical Committee of the Behavioural Science Institute of Radboud University Nijmegen, the Netherlands, approved this study.

### Instruments

#### Interpretation Task

The interpretation task consisted of 16 open-ended scenarios, each scenario contained 5 short sentences. All scenarios were ambiguous, such that they could be interpreted in a positive, neutral, or negative way. The set of 16 scenarios was adapted and translated into Dutch from existing materials [14, 15, 17, 31, 32]. The 16 scenarios were divided into 2 categories; 8 spider threat-related scenarios and 8 social threat-related scenarios (see Table 1 for sample scenarios).

**Table 1:** Sample stories and sample endings of the two categories of the interpretation task

Scenarios	Sample ending
Spider threat-related scenario: “Vacation” You are free from school and the weather is very nice. You have helped your mother in the garden today. It is half past nine and you have to go to bed. You brush your teeth and walk into your bedroom. In the corner of your room you see a cobweb…	‘oh no, there is a huge hairy spider in that cobweb that wants to bite me’
Social threat-related scenario: “Birthday” Today is your grandmother’s birthday. It is already busy when you arrive. You give your grandmother a present. Everybody is watching when grandma opens the present. Then all of a sudden someone laughs really loud…	‘everyone thinks the present is stupid.’

The children were asked to read aloud two blocks of eight scenarios on a computer screen and to imagine themselves as the central character in each scenario. For each scenario, they were asked to think of approximately two sentences to end the story. Their own endings were recorded with a microphone, to be transcribed and scored later. They had a short break between the blocks. Scenarios were presented in random order, but each block consisted of four scenarios from each category.

The second and third author, blind to the children’s’ level of fear, coded the interpretations. The endings were scored on a 5-point scale, ranging from 1 = positive, 2 = slightly positive, 3 = neutral, 4 = slightly negative, to 5 = very negative [see also 17]. The higher the score, the more the child tended to interpret the scenario as negative. If a scenario ending was unclassifiable, for instance due to unclear recording, the raters scored the scenario as missing. First, 25% of the scenarios were coded by both raters independently of each other (4.6% missing). The raters agreed on 83% of the ratings and had a kappa of 0.90, which suggests a high agreement [33]. Next, the third author coded all remaining scenarios.

#### Spider Anxiety and Disgust Screening for Children [SADS-C; 25]

The SADS-C is a self-report questionnaire that measures responses to four spider-related statements on a 5-point Likert scale. The four statements address fear of spiders, physical reactions, avoidance, and disgust. Internal consistency and test–retest reliability are satisfactory (*α* = 0.88, *r* = 0.91) [25].

#### Social Anxiety Scale for Children: Revised [SASC-R; 34]

The SASC-R is a self-report questionnaire to measure social anxiety symptoms. The SASC-R consists of 18 items on a 5-point Likert scale ranging from ‘never’ to ‘always’. Eight items of this questionnaire measure fear of negative evaluation (e.g., ‘I worry about what other children say about me’). Six other items of the SASC-R measure social avoidance and distress in new situations, for example ‘I get nervous when I talk to kids I don’t know very well’. The other items of this questionnaire were designed to measure social avoidance and distress in general (e.g., ‘I am quiet when I’m with a group of kids’). The validity and reliability of the SASC-R are satisfactory [34].

#### Behavior Avoidance Task (BAT)

This task was used to assess the children’s avoidance behavior when confronted with a tarantula skin, which they believed to be a real, living spider. The task was identical to the BAT described by Klein et al. [35]. BAT performance was scored on an 8-point scale: The child was asked to enter a room in which a covered box containing a tarantula was located. The child was asked to stand on a mark visible on the ground 3 m away from the box. The experimenter explained to the child that a real tarantula was in the box. The child’s task was to approach the box as closely as he or she liked. The experimenter stressed that the procedure was not a competition, and if the child did not want to approach any further, he or she could say so immediately. The experimenter then uncovered the box and the child was told to look at the tarantula. The child received points initially for each meter traversed towards the box (1 m closer to the box: 1 point, 2 m: 2 points, and next to the box: 3 points). The child was then asked to put a hand on the box for > 10 s (4 points), and then to lift the box (5 points). After putting down the box, the child was then asked to open the lid (6 points), and to put one hand in the box (7 points). The last step was to touch the tarantula with one hand (8 points). If a child failed to take any particular step, or wanted to stop, the last completed step was recorded as the BAT score. During the child’s performance of the BAT, the experimenter knew neither the child’s SADS-C scores nor its interpretation task or memory task scores.

#### Social Speech Task (SST)

The SST was used to get an indication of the children’s levels of anxiety during an anxiety-provoking situation. As the literature is inconclusive as to whether socially anxious individuals behave differently from non-anxious individuals during anxiety-provoking situations [22], we decided to measure self-reported anxiety [see also 26]. The child was asked to indicate anxiety on a 0- to 10-point Likert scale ranging from “not anxious at all” to “very anxious”, right *before* the instructions of the SST, when the child was relaxed and unaware of having to perform the SST. The child was asked to indicate anxiety again directly following the SST [13, 16].

The SST itself consisted of a 2-min impromptu speech in front of a camera. The children were told that their speech would be recorded and that adults would rate their video afterwards. The children were asked to speak about any topic, and the research assistant gave a few examples of subjects to talk about. After the instructions, the child was asked to stand straight up facing the camera, and the assistant sat on a chair behind the child. When the child fell silent for > 10 s, the assistant gave a standardized prompt. There was a maximum of three prompts, each of which was given after 10 s of silence. Two children indicated that they did not want to proceed with the task, and the task was terminated immediately.

### Procedure

The testing was divided into two sessions in which the children performed the tasks and filled in the questionnaires individually, accompanied by a trained research assistant. In the first session, the children performed the spider BAT. In the second session, the children first performed the interpretation task followed by the SST. Finally, they filled in the questionnaires.

## Results

### Descriptives

Not every child was able to perform all measures because of technical problems with the tasks or they did not finish the session (interpretation task: *n* = 4; SADS-C: *n* = 2; SST: *n* = 3). We decided to analyze all available data.

The mean sum score on self-reported spider fear as measured with the SADS-C, was 11.6 (*SD* = 5.3) and the mean sum score on self-reported social anxiety as measured with the SASC-R was 15.7 (SD = 4.8). The mean score on the interpretation task was 2.5 (*SD* = 0.3) for spider threat-related scenarios, and 2.6 (*SD* = 0.4) for social threat-related scenarios. The mean approach score on the spider-BAT was 5.2 (*SD* = 2.5; min = 0, max = 8). Finally, the mean sum scores on the self-reported anxiety scale of the SST were 5.8 (*SD* = 7.5) before the instructions of the SST, and 9.1 (*SD* = 10.39) after the SST. A difference score was calculated by subtracting the pre-anxiety measurement from the post-anxiety measurement (Social SST-change score), which resulted in a mean score of 3.51 (*SD* = 6.35), indicating that the children experienced more anxiety directly following the SST than before they were aware of having to perform the SST, *t*(137) = 6.5, *p* < 0.001. There were no significant differences between age or gender on any measures, except for the fact that girls (*M* = 2.49, *SD* = 0.30) scored marginally higher on the spider-interpretation bias scores than boys (*M* = 2.39, *SD* = 0.29), *F*(1,136) = 3.46, *p* = 0.065, and older children approached the spider more closely than younger children (*r* = 0.19, *p* < 0.05). We therefore decided to control for age and gender in the analyses reported below.

### Correlations

As expected, the correlation between SADS-C scores and BAT scores was significant with a large effect size, *r* = − 0.52 (*p* < 0.001): children who reported more fear of spiders avoided the spider more. Likewise, the correlation between the SASC-R and the social SST-change score was also significant with a medium effect size, *r* = 0.38 (*p* < 0.001): children who reported a higher level of social anxiety on the SASC-R also reported higher anxiety on the SST. Self-reported social anxiety was also significantly related to self-reported spider fear with a medium effect size (*r* = 0.25, *p* = 0.004).

As expected, based on Klein et al. [17], the spider-interpretation bias scores correlated significantly with self-reported spider fear (*r* = 0.26, *p* = 0.003), and with avoidance on the BAT (*r* = − 0.28, *p* = 0.002) and with a similar medium effect size as reported by Klein et al. [17]. The spider-interpretation bias scores correlated neither with self-reported social anxiety (*r* = 0.08, *p* > 0.1), nor with the social SST-change score (*r* = 0.04, *p* > 0.1). Unexpectedly, the social-interpretation bias scores did not correlate significantly with self-reported social anxiety (*r* = 0.04, *p* > 0.1). However, social-interpretation bias scores correlated significantly with the social SST-change score (*r* = 0.25, *p* = 0.004), again with a similar medium effect size as reported above and reported by Klein et al. [17]. As expected, the social-interpretation bias scores correlated neither with self-reported spider fear (*r* = − 0.04, *p* > 0.1), nor with avoidance on the BAT (*r* = 0.11, *p* > 0.1). This indicates that children with self-reported symptoms of spider fear and a high avoidance of spiders on the BAT displayed negative interpretation biases only for materials related to spider threat. Children with higher anxiety on the SST displayed negative interpretation biases only for social threat, but self-reported social anxiety symptoms were not significantly related to a negative interpretation bias towards social threat.

### Regression Analysis

In order to address research goal three, to predict spider fear-related behavior measured by the BAT, we used a hierarchical regression analysis with BAT scores as the criterion. SADS-C scores, SASC-R scores, spider-interpretation bias scores and social-interpretation bias scores were used as predictors (see Table 2). We also included Age and Gender in step 1 of the regression, in order to control for these variables. The model with age and gender was marginally significant, *F*(2,132) = 2.57, *p* = 0.08, and explained 3.8% of the variance in BAT behavior. For this first regression, Gender was a significant predictor, indicating that girls approached the spider more closely than boys. After the second step, when we also included the predictors of interest, the model reached significance, *F*(6,128) = 11.00, *p* < 0.001, and explained 34.0% of the variance in BAT behavior. This model was significantly better than the first model, *F*(4,128) = 14.67, *p* < 0.001. For this second regression, age, gender, SADS-C scores, and spider-interpretation bias were significant predictors (see also Table 2). Thus, self-reported spider fear and spider-interpretation bias scores predicted unique variance in fear-related behavior on the BAT. Interpretation bias related to spiders was thus able to predict unique variance above and beyond the variance predicted by self-reported spider fear. Additionally, age and gender were significant predictors in the model, indicating that older children and girls approached the spider more closely than younger children and boys. Furthermore, the measures related to social anxiety were all non-significant, indicating content-specificity.

**Table 2:** Hierarchical regression analyses predicting BAT and social SST scores from age, gender, questionnaire scores, and interpretation bias scores

Criterion variable	Step	*R* ^2^	*R*^2^ change	Predictor	Standardized regression coefficients (*β*)
BAT-score	1	0.04			
				Age Gender	0.06 0.18*
	2	0.34	0.30**		
				Age Gender	0.23* 0.14*
				SADS-C	− 0.40**
				SASC	− 0.11
				Spider-interpretation bias	− 0.16*
				Social-interpretation bias	0.10
SST-score	1	< 0.01			
				Age Gender	− 0.01 − 0.03
	2	0.22	0.22*		
				Age Gender	− 0.07 − 0.07
				SADS-C	0.15^+^
				SASC	0.34**
				Spider-interpretation bias	− 0.03
				Social-interpretation bias	0.25*

^+^p < 0.1, *p < 0.05, **p < 0.001, standardized *β* coefficients are reported

In order to predict anxiety during the SST, we repeated the regression analysis, but now included the social SST change-scores as the criterion (see Table 2). The model with age and gender did not reach statistical significance (*p* > 0.1). After the second step, when we included the predictors of interest, the model was significant, *F*(6,125) = 6.01, *p* < 0.001, and explained 22.4% of the variance in social SST-change scores. This model was significantly better than the first model, *F*(4,125) = 8.97, *p* < 0.001. For this second regression, SASC-R scores and social-interpretation bias scores were significant predictors (see also Table 2). Thus, self-reported social anxiety as measured with the SASC-R, and social-interpretation bias scores predicted unique variance in social SST-change scores on the SST. Interpretation bias related to social situations was thus able to predict unique variance above and beyond the variance explained by self-reported social anxiety. Furthermore, the measures related to spider fear were all non-significant, indicating content-specificity. It should be noted, however, that the SADS-C was a marginally significant predictor (*β* = 0.15, *p* = 0.071).

## Discussion

We investigated whether children with varying levels of spider fear and/or social anxiety display interpretation biases and whether these biases are content-specific. Furthermore, we evaluated the extent to which responses to a behavioral task were predicted independently by self-reported fear and interpretation biases. The first goal was to compare the findings of the current study to an earlier study reported by Klein et al. [17]. Consistent with our hypothesis and the results of Klein et al. [17], we found that children with higher self-reported spider fear and avoidance behavior on the BAT displayed an interpretation bias for spider threat-related stimuli only. This indicates that children with higher levels of spider-fear display an interpretation bias for content-specific materials only. The second goal was to test whether children with higher levels of self-reported social anxiety and a higher anxiety score during the SST would show more negative interpretations of ambiguous social threat-related scenarios only, and not of spider threat-related scenarios. As expected, children with higher anxiety on the SST displayed an interpretation bias for social threat-related stimuli only. However, unexpectedly, children’s self-reported social anxiety was not significantly related to social-threat related interpretation bias. The third goal of this study was to compare whether self-reported fear and the interpretation task each uniquely predicted responses during the behavioral tasks. As expected, we found that self-reports and the interpretation task each predicted unique variance in responses to fear-related stimuli. This was true for both spider-fear related behavior as well as levels of anxiety during a speech task. This means that both direct and indirect measures were necessary for an optimal prediction of fear-related stimuli in children. This also means that the inclusion of the interpretation task, besides the questionnaire, predicted even more variance in fear-related stimuli. Furthermore, we found this relation to be specific for each fear; self-reported spider fear and a spider-threat related interpretation bias were the only significant predictors in explaining avoidance behavior towards a spider, and self-reported social anxiety and social-threat related interpretation bias were the only significant predictors in explaining anxiety during the SST. It should be noted, however, that self-reported spider fear was a marginally significant predictor in the regression analysis predicting anxiety during the SST, but the contribution relative to the other predictors was small. Hence, this not only strengthens the evidence for the existence of content-specificity of interpretation processes in childhood anxiety, but also the independent role of interpretation biases in predicting measures related to behavioral tasks.

These results are in line with findings reported by several other studies on the content-specificity of interpretation biases for threatening information in children with anxiety [15, 17, 18]. These results are also consistent with findings from the broader childhood anxiety literature, supporting cognitive-specificity models in children and adolescents when examining automatic thoughts [36–38] and with dual processing models [e.g. 24]. In addition, there is a notable similarity between the results of the present study and those demonstrating specificity in interpretation biases in anxious adults [39]. Taken together, the data suggest continuity in content-specificity across the course of development, in line with cognitive theories of psychopathology. It may be that patterns of cognitive functioning associated with emotional states are established at a relatively early age, and tend to continue into adolescence and adulthood.

Clinically, these findings regarding content-specific interpretation biases related to spider fear and social anxiety might be used to improve treatments for anxious children. To date, most childhood Cognitive Behavioral Treatment programs focus on generic anxiety management without distinguishing between specific anxiety disorders. As a result, approximately 40% of treated children still meet criteria for a clinical disorder after treatment [3]. This study shows that it may be important to focus on individual anxiety disorders, and also on processes that are relevant for the child’s individual anxiety disorder.

The mixed results in the literature regarding the content-specificity of interpretation biases related to social anxiety warrant further discussion. While there are a few studies that failed to find evidence of content-specific interpretations in children with social anxiety [13, 14], we have found some evidence for the content-specificity of interpretation biases in children with varying levels of social anxiety (in line with [15, 18]). It is difficult to compare the studies because of differences in the characteristics of the children that were studied. The study by Muris et al. [14] for instance, studied an analogue sample, while two other studies investigated clinically anxious samples [13, 18]. Another difficulty in comparing the studies is the different measures that were used to study interpretation bias. In-Albon et al. [13], for instance, used an indirect measure, whereas two other studies used relatively direct measures [14, 15] including different stimuli, such as pictures, words, or scenarios [see also 19].

One of the explanations of our unambiguous finding of the existence of content-specificity in children’s interpretation biases might be that we compared a physical threat (spider fear) to a social threat (speech fear). A few studies in both adults and children have found evidence suggesting that feared outcomes are organized into two major dimensions; namely, concerns about physical threat or harm, versus concerns about social threat or negative evaluations [19, 40]. This would mean that the specificity of interpretation biases is not organized around the different anxiety disorders, but that anxiety disorders can be clustered into domains of fear, i.e. physical threat versus social threat. Note that there may exist more than two dimensions of fear, for example separation fear may be a different domain of fear, in children as well as in adults [41]. However, the point we want to make is that studying specificity of interpretation bias around classifications of anxiety disorders may not be optimal, as for example generalized anxiety disorder is characterized by worries about physical as well as social threats. In line with this point, levels of social anxiety and spider fear showed only a weak significant correlation.

Another possible explanation of our findings is that we included behavioral tasks in addition to self-reports. In this study, we found that self-reported social anxiety and the social-threat related items of the interpretation task were unrelated, whereas the interpretation task correlated significantly with levels of anxiety during the SST. These differences in findings might be due to the selected scenarios. For spider-fearful children, the scenarios they encountered were specific to their fear, because fear of spiders is more homogenous; all spider fearful children are afraid to encounter a spider. We cannot be sure that this was also the case for the social scenarios, as social situations are more heterogeneous, for example, children might be afraid of interactions with their peers, or with adults, at school or new situations. It might have been the case that the social scenarios did not perfectly match the children’s social worries, and that the social scenarios therefore did not correlate significantly with self-reported social anxiety. Moreover, the relation between interpretation bias and self-reported fear might be less visible in anxious children from a community sample than in clinically anxious children. For example, Schneider et al. [32] only found interpretation biases in children of parents with an anxiety disorder after these children were primed with a video about their mothers’ anxiety. They concluded that at risk children only show biases in interpretation when their anxiety-related schemata are activated. As our SST can be seen as a type of priming (the SST induces state anxiety), this might be the reason for the significant correlation between the SST and the social-threat related stimuli of the interpretation task. Clearly more research is needed into the specificity of interpretation biases that addresses the issues raised above. Future studies, should, for instance, compare analogue samples to clinically anxious children, using different formats of studying interpretation biases at the same time, include behavioral measures, and also focus on possible differences between physically orientated and socially orientated anxiety disorders.

This study has several limitations. First, the study was limited to children with varying levels of spider fear and social anxiety, and relatively few boys were included. Second, we did not include a diagnostic interview to find out whether some of the children experienced an anxiety disorder. Finally, because the published results are inconclusive as to whether socially anxious individuals behave differently from non-anxious individuals during anxiety-provoking situations [22, 42, 43], we decided to measure self-reported anxiety before and after the SST, instead of using a measure of actual behavior in this study. Even though the children were confronted with a real life phobic stimulus, and were not only asked to think about how they would feel in such a situation, but had to actively participate in this anxiety-provoking situation, it would be worthwhile to include more behavioral aspects of social anxiety in future studies.

In summary, we found that the interpretation task can be a useful instrument for assessing the specificity of cognitive processes in children with varying levels of spider fear and social anxiety. The present results support cognitive models in youth that argue for the specificity of cognitive content associated with different disorders [8]. The results are consistent with current classification systems for childhood mental disorders [44] as well as with the two-dimensional factor models [19, 38]. Clinically, this insight into the presence of interpretation biases in anxiety disorders might be used to improve treatments for anxious children by targeting content-specific interpretation biases related to individual disorders.

## Summary

Cognitive theories highlight the importance of cognitive processes in the onset and maintenance of anxiety disorders. This study investigated the role of self-reports and behavioral measures in studying interpretation biases and their content-specificity in children with varying levels of spider fear and/or social anxiety. In total, 141 selected children from a community sample were asked to complete an interpretation bias task with scenarios that were related to either spider threat or social threat. Children also completed the SADS-C, the SASC-R, and performed a Behavioral Assessment Test (BAT) related to spiders, and a SST. Specific interpretation biases were found; children with higher levels of self-reported spider fear and more avoidance behavior on the BAT showed more negative interpretations of ambiguous spider threat-related scenarios, but not of social threat-related scenarios. Likewise, children with higher levels of self-reported anxiety during the SST, showed a specific interpretation bias for social threat, but not for spider threat. Unexpectedly, there was no significant relation between self-reported social anxiety and an interpretation bias related to social threat. Furthermore, both spider-related interpretation bias and self-reported spider fear predicted unique variance in avoidance behavior on the BAT. Both social-threat related interpretation bias and self-reported social anxiety predicted anxiety during the SST. These findings support the hypothesis that fearful children display cognitive biases that are specific for fear-relevant stimuli. They also emphasize the importance of using behavioral measures in addition to questionnaires, especially when using more indirect measures to study the more automatic aspects of cognitive processing. Clinically, this insight might be used to improve treatments for anxious children by targeting content-specific interpretation biases related to individual disorders.
